# Ten simple rules for navigating AI in science

**DOI:** 10.1371/journal.pcbi.1013259

**Published:** 2025-07-18

**Authors:** Aidan Crilly, Alice Malivert, Andreas Christ Sølvsten Jørgensen, Claire E. Heaney, Gema I. Vera Gonzalez, Marcus Ghosh, Manolo Fernandez Perez, Mikael M. Mieskolainen, Mohammed Azzouzi, Zhenzhu Li

**Affiliations:** 1 I-X Centre for AI In Science, Imperial College London, London, United Kingdom; 2 Centre for Inertial Fusion Studies, The Blackett Laboratory, Imperial College London, London, United Kingdom; 3 Department of Bioengineering, Imperial College London, London, United Kingdom; 4 CIRAD, UPR HortSys, Saint-Pierre, La Réunion, France; 5 HortSys, CIRAD, Université de Montpellier, Montpellier, France; 6 Department of Mathematics, Imperial College London, London, United Kingdom; 7 Department of Earth Science and Engineering, Imperial College London, London, United Kingdom; 8 Department of Mechanical Engineering, Imperial College London, London, United Kingdom; 9 Department of Electrical & Electronic Engineering, Imperial College London, London, United Kingdom; 10 Department of Life Sciences, Imperial College London, London, United Kingdom; 11 Real Jardín Botánico (RJB), CSIC, Madrid, Spain; 12 Department of Physics, Imperial College London, London, United Kingdom; 13 Department of Chemistry, Imperial College London, Molecular Sciences Research Hub, London, United Kingdom; 14 Department of Materials, Imperial College London, London, United Kingdom; Carnegie Mellon University, UNITED STATES OF AMERICA

## 1. Introduction

**Artificial Intelligence** (AI) promises to have a huge impact on science in the years to come. For a domain expert within a scientific discipline, it can, however, be hard to navigate the vast body of literature surrounding AI techniques and the latest developments. As scientists using AI in diverse fields, from plant biology to neuroscience and physics, we have faced these and similar questions over the years. In this paper, we share some of the key aspects of our learning journeys.

Over recent decades, all scientific disciplines have been transformed by advances in computational methods, with AI methods now coming to the fore. An example of the success of AI includes the defeat of chess champion Garry Kasparov in 1997 by IBM’s computer “Deep Blue”, which was capable of examining 200 million moves per second [1]. Deep Blue, a text-book example of Good Old-Fashioned AI (GOFAI) [2], used a brute-force search strategy combined with hand-crafted evaluation functions and deterministic rules. The search strategy involved alpha-beta pruning (which reduces the number of nodes evaluated in a search tree by pruning branches that do not affect the outcome) and iterative deepening (which repeatedly performs depth-limited searches in a search tree but increases the depth with each iteration). This led to an ability to generate a large number of moves ahead, routinely 14; however, for moves of interest, it could generate up to 20 moves ahead [3]. Although beating Kasparov was significant, it took millions of dollars and years of staff time, and the algorithm could not easily be applied to applications beyond chess [4]. Offering an alternative paradigm, machine learning has since risen to the attention of the community, with the potential for a machine to learn from data rather than relying on humans to hand-code rules.

Notable successes of AI, in the form of machine learning, show how it is making an impact in science. The first example is the prediction of the 3D structure of proteins by a **deep learning** model called AlphaFold [5]. This breakthrough has given scientists insight into protein function, which will enable targeted drug delivery. Another example is controlling the instabilities that occur within plasmas during fusion reactions by a deep **reinforcement learning** model, which has enabled a plasma to be sustained long enough to see the first-ever net energy gain from fusion [6]. Finally, aside from pervading many aspects of modern daily life, **large language models** (LLMs), such as ChatGPT [7], are now being applied in scientific contexts, for example, to generate neuroscientific models [8] and to perform analytical calculations in theoretical physics [9].

Our paper is primarily aimed at early-career STEM researchers, with some coding experience, who hope to apply AI methods to their scientific domain. With this in mind, we have kept our rules light on technical detail but include a detailed glossary and didactic references to help readers dive deeper into the topic and apply these rules in practice. Readers with more expertise, in applying AI to scientific problems or machine learning, may find our rules a useful framework for teaching. Thus, we hope that readers, regardless of their level of expertise, will find value in the paper and benefit from revisiting it at different stages of their journey into the field of AI.

**Fig 1:**
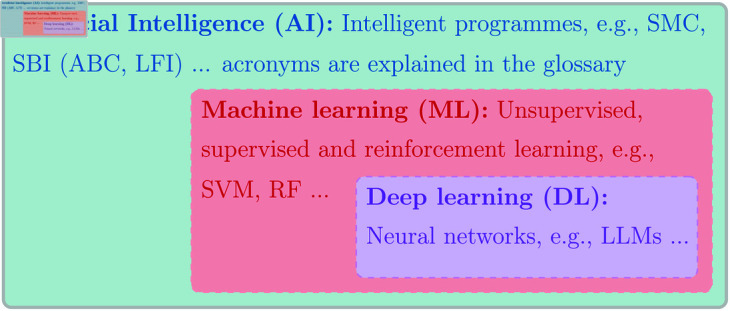
In this paper, we use the term AI in its broadest sense. It thus covers a wide-ranging set of algorithms, including machine-learning and deep-learning techniques as subcategories, as illustrated here. We include concrete examples, of which most are discussed in the text, and all are explained in the glossary (Table A.1) in Appendix A in S1 File. Beyond signaling the relation between central terms in AI, the figure is hence intended to complement the glossary by sorting and clustering terms. SMC: **Sequential Monte Carlo**. SBI: **Simulation-based inference**. ABC: **Approximate Bayesian Computation**. LFI: **Likelihood-free inference**. SVM: **Support vector machine**. RF: **Random forest**. LLM: **Large Language model**.

Especially since the recent surge of LLMs, the term AI is hotly debated [10]. While **generative algorithms** for text and image data, such as LLMs, might thus immediately come to mind when mentioning the term AI, we note that we will be using the term in its broadest sense here. Hence, AI covers a variety of algorithms ranging from **machine-learning** (ML) tools to statistical methods, while machine learning itself covers a wide variety of methods, including but not limited to neural networks and deep learning [11]. Other sources might use the term AI in a narrower sense, but none of the takeaways in this paper are method or programming language-specific. See Fig 1 for an illustration of the relationship between some of the AI methods we mention here.

The rules in this paper are ordered according to the flow of scientific exploration. First, we discuss how to frame the scientific problem and identify suitable AI algorithms. Then, we turn to rules associated with coding before discussing ethical considerations, including the explainability, interpretation and robustness of the results.

Introducing ethical considerations towards the end of the paper provides a suitable overview of the underlying technical background required to contextualise these issues. Meanwhile, the opposite holds true too: Addressing ethical considerations is fundamentally important to fully appreciate the technical challenges, particularly when considering reproducibility. Not all AI methods produce easily explainable or interpretable outputs, and not all AI models are robust and able to generalise to new scenarios. While interpretability and generalisability already pose challenges in traditional scientific endeavours, these issues can make it especially difficult for many AI methods to achieve the level of reproducibility required in science. Understanding these aspects is vital (e.g., [12]), and although a full discussion of reproducibility and explainability is beyond the scope of this paper, we hope that our ten rules equip readers with the tools and awareness to identify and address limitations in their AI models.

**Fig 2:**
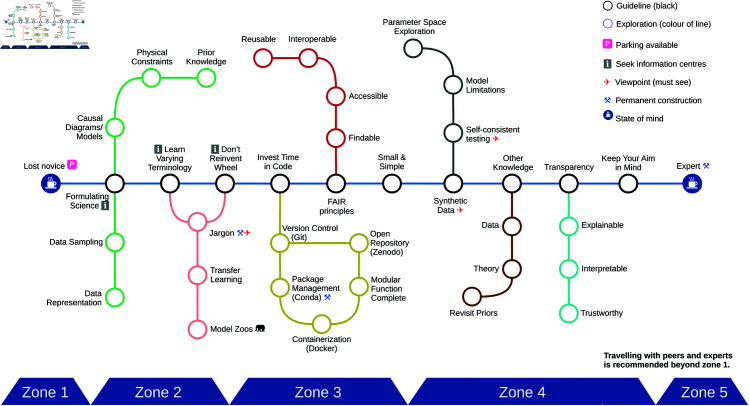
Info-graphics summarising how to navigate the field of AI in the form of a tube (metro) map. The main (blue) line denotes the rules (1-10) presented in this paper, while the other lines (green, pink, yellow, grey, red, brown and cyan) dive deeper into each of the guidelines by providing key terms associated with each rule. The aesthetic choices behind the info-graphics imply that we draw on metaphors to help make our rules memorable. For instance, we indicate that concrete tools might change (“under construction”). Similarly, to help readers avoid pitfalls, we highlight a few points where we encourage the reader to pause despite the urge to quickly apply AI to real-world data (“viewpoints”). Tracks bending in either direction indicate that some of the terms and concepts reappear in later or earlier rules. The rules in this paper are ordered according to the flow of scientific explorations: Framing the scientific problem and finding AI algorithms (Zone 2), coding (Zone 3), and testing and interpreting results (Zone 4). We hope it helps the reader to navigate on the journey from novice (Zone 1) to expert (Zone 5) in the topic. The silhouettes of the elephant and coffee mug are both taken from Wikimedia Commons and are in the public domain, while the remaining pictograms, including the hammer and pick, the parking sign and the aeroplane, are Unicode characters (U+2692, U+1F17F and U+2708).

In the supplementary material (Appendix A in S1 File), we provide a glossary containing key terms highlighted in bold in the text, while Fig 2 shows a stylised road map of our rules. We also refer the interested reader to other info-graphics illustrating groupings of AI algorithms and techniques, e.g., from the software package scikit-learn, from a review of machine-learning approaches used in fluid dynamics (cf. Fig 1 in [13]) and from a survey of AI approaches used in anesthesiology (cf. Fig 2 in [14]).

## Rule 1: Frame your scientific question

Navigating AI for science requires that you first frame your scientific questions and consider whether AI techniques can help provide answers. Having a clear and objective scientific question will help you find an optimised approach to selecting a suitable AI technique. The type and range of behaviour captured by any real-world data that you might set out to explore also needs to be considered, as it might affect your choices. Some AI methods might require large amounts of data. Is it reasonable, given your data, that an AI model can answer your scientific questions?

Since your data determines which algorithms are applicable and guides your scientific inquiry, you should thoroughly understand your data and consider topics such as **data cleaning**, data preparation and formatting before diving into specific AI algorithms. For more information on data processing, we recommend other papers from the Ten Simple Rules series [15,16].

Are you aiming for algorithms that can provide predictions or emulate data rather than a mechanistic understanding of the underlying system? If your scientific question involves the collection of a **labelled dataset**, you could focus on **supervised learning** approaches, such as **classification** or **regression**. When dealing with unlabelled data, on the other hand, you should turn to **unsupervised learning** methods, such as **clustering** or **dimensionality reduction**.

Do you have an understanding of the mechanisms that underlie your system? In that case, you might want to go down the route of Bayesian statistics and **causal inference** (see also Rule 8). You should then consider designing your model as a causal diagram [17]. This approach allows you to conceptualise the relationships among the variables you aim to estimate, identify potential **confounding factors**, and predict the outcomes of interventions on different variables [18].

No matter which path you take, it is crucial to scrutinise what constitutes a *good* model based on your data and your scientific objective (see also Rule 6). Which meaningful performance measures can you use, and what do they really tell you? Standard measures, such as **accuracy**, are on their own seldom sufficiently informative.

## Rule 2: Learn the varying terminology inside and outside your field

When starting to work with AI, a major challenge is to become familiar with its extensive vocabulary, which can seem opaque at first. With this in mind, we include various technical terms throughout the paper to give novices a starting point for diving into AI and to assist more experienced practitioners to link each rule to concrete examples.

Often, the same technique or model can receive different names or labels in different fields or even in the same field at different points in time. For example, **Sequential Monte Carlo** (SMC) are a set of methods that are also often referred to as particle filters. The two terms can hence be used synonymously.

Conversely, some terms can hold several meanings. For instance, in different AI contexts, **bias** can be used for: (1) **bias-variance trade-off**; (2) **a biased dataset** (bad); (3) **inductive or learning bias** (good); (4) **bias parameters** of a **neural network** (see Fig A.1 in Appendix A in S1 File).

Mastering field-specific terminology will enhance your communication with colleagues, while mastering the terminology outside of your field will allow you to access material that helps you think outside of the box (cf. Rule 3). A solid grasp of terminology will also help you explore the literature in your field and other fields more effectively.

There are many useful, readily available resources on AI, ranging from online courses to papers (e.g., [19]). However, in order to gain knowledge on varying terminology or such material, you may need to go beyond generic AI courses and reading papers. Seminars and reading groups can be challenging to follow, but they will help you absorb jargon through immersive learning, much like learning a new language. If you are in an academic environment, suitable reading groups might already exist. If not, consider establishing these opportunities yourself. Learning with peers fosters accountability and boosts your progress.

## Rule 3: Do not reinvent the wheel

A wide range of packages exist that allow you to implement machine-learning methods with just a few lines of code (cf. e.g., scikit-learn [20]). Similarly, other researchers might already have addressed research questions related to your own.

To leverage this, you should explore methodologies and solutions, including those from other disciplines, to find the ideal tool for your situation. Be curious, read the literature from your field and other fields, as discussed in Rule 2, and explore **model zoos** and **foundation models** to build on existing work [21]. This ties in with our recommendations from Rule 2: Engaging with AI experts and peers, even from unrelated fields, can be very helpful as they can provide valuable insights and recommend algorithms or solutions. It will save you from unfocused and exhausting searches of the literature and maybe start collaborations, further advancing your research, as AI is a tool used across disciplines and thus provides a common ground for scientific fields.

Using models and packages developed by others can be particularly useful when relying on experimental data for training. This might seem like a daunting ask at first, as recent high-performing models, and in particular transformers such as the Segment Anything Model, are trained on vast amounts of data. Many scientists, especially experimentalists, cannot afford either the data collection, data annotation or the computational costs associated with training such models. However, techniques such as **transfer learning** and **data augmentation** schemes (cf. Rule 7) can help make AI more accessible while conserving high performance.

## Rule 4: Invest time in your code

You should invest time in maintaining your code throughout your project. This may feel like time away from research, but it will improve the quality and reproducibility of your work and save you time in the long run. We recommend following the Good Research Code Handbook [22]. We summarise some of its key points below.

At the start of your project, you should set up a repository for version control (e.g., using Git) to back up your code, track changes, and allow for easy sharing. Tools for version control can also be used to make collaborations easier.

You might, for good reasons, decide to build your algorithms from scratch. However, many research projects nowadays have shifted to combining and building upon existing packages as mentioned in and Rule 3 [23]. While this can accelerate your project, it comes with its own pitfalls. Updates to external packages might affect or break your code, and you might be working on different projects that each require different versions of certain packages. To address this issue, it is advisable to set up a virtual environment using tools, such as Conda or Docker, to track which packages and versions you are using (cf. **containerization)**.

Throughout your project, you should write your code using **modular functions** complete with documentation describing each function’s arguments and outputs; regularly push your changes to Git; and keep track of your assumptions and settings.

To develop your coding skills, consider following online courses, e.g., the Python Data Science Handbook, participating in competitions, e.g. Kaggle, or contributing to open source projects.

## Rule 5: Bear in mind the FAIR principles

In order for AI to be scientifically useful, it needs to be scrutinised to the same standard as other scientific findings. This requires that the scientific community can reproduce your AI model or, indeed, further develop it, leading to greater understanding and interoperability (see also Rule 9). These ideas are summarised in the **FAIR** (Findable, Accessible, Interoperable, Reusable) principles [24,25]. Originally designed for data sharing, the “FAIR principles” have now been widely adopted in research software engineering, code development and research data publication.

To ensure reproducibility, your code should be as open-source as possible. Tools for this purpose have been discussed in Rule 4. Many online platforms also exist that are useful for organising your projects by providing lifecycle solutions for research projects. For example, Zenodo and OSF are widely adopted for sharing projects by providing storage for data files, text files for text-mining purposes, codes, and supplementary materials. They also provide an affiliate indicator so that visitors can trace contributors and projects to your collection to discover even more related research. In addition to sharing your code, be prepared to make your training data available (if feasible) so that other researchers can experiment with other models using the same training data. For machine-learning applications, also consider sharing your model and its weights so that others can run your model without having to train it.

**Machine Learning Operations** (MLOps), are a set of practices for machine-learning workflows and deployments (e.g., monitoring performance) to bring your machine-learning research to bear on a broader set of real-world applications [26]. MLOPs tools are packages that can help you integrate these practices (e.g., Weights & Biases for monitoring your model development). They can act as your lab book and are a great way to ensure a scientific workflow is applied in your machine-learning endeavour.

Bearing in mind the FAIR principles during your use of AI in science will be of great value to both the community and yourself by enhancing the transparency and reproducibility of your research results and helping democratise AI to a wider scope of researchers.

## Rule 6: Start small and simple

You may be tempted to use the latest, state-of-the-art method. However, for many questions, simpler models will suffice and will be faster to fit and interpret. Indeed, whether there is anything to gain from more complex algorithms will heavily depend on your problem and your data.

We suggest using the following approach: First, establish a baseline to evaluate how well your problem could be solved by a naive approach, such as randomly guessing the answer or always guessing the most likely answer. This is also a recommended practice by scientific journals such as Digital Discovery. In the checklist of its code review, the reviewers will also review your code against the performance of baseline models, such as **random forest**, **support vector machines** or **Extreme Gradient Boosting search** (xGBoost). This provides you with a reference against which to evaluate other approaches. Having established a baseline, start with simpler approaches and then increase complexity. For instance, when facing a classification task, try non-deep learning approaches first. These are quick to implement and test via **libraries** such as scikit-learn [20], and they may already perform well enough for your needs. If not, you could try increasingly complex **neural network** models. Initially, these models may perform worse than your non-deep learning method or even your baseline. However, by adjusting their **hyperparameters**, you should be able to improve performance. You can “tune” these hyperparameters by hand, by changing values, and **training** and **testing** models, or by using a tuning tool such as Ray Tune [27].

In the same spirit, you may not want to address all aspects of your research problem simultaneously. Start by aiming to recover key trends or global properties of your system.

## Rule 7: Start with synthetic data

If your model fails to perform well, two possible explanations exist. Either the problem lies in your model (e.g., your code, your choice of hyperparameters), or your data may be insufficient to answer your research question. To distinguish between these two scenarios, you should ensure that your code can produce a model with reasonable performance on **synthetic data**, i.e., data that are generated using a model. In the simplest case, this could be points drawn from Gaussian/normal distributions. However, in other cases, more realistic data may be required.

As synthetic data are generated via a model, all aspects of the data are under your control. This means that you can use synthetic data to explore the performance of your model in various scenarios. For example, how does it perform with increasing noise or smaller datasets? Moreover, synthetic data allows you to make comparisons and tests that are completely self-consistent and transparent. Consider the case where your model is a dynamic or statistical description of a phenomenon, and you are aiming to infer the values of your model parameters, e.g., using Bayesian inference. As a simple example, consider a linear fit. In a fully self-consistent comparison, you would first create the synthetic data by choosing a set of parameter values and generating model output using a version of the *same* model that includes adequate noise. Subsequently, you use your inference framework to see how well you can recover the original parameter values. Since you know which parameter values were used to generate the synthetic data, you can measure the performance of your model in absolute terms, catching otherwise deceptive errors. Thus, synthetic data are an invaluable diagnostic tool even when your model does not seem to perform poorly. This notion also goes hand in hand with a suitable definition of the performance metrics as discussed in Rule 1 and other quality checks (see also [28]).

Another way of creating synthetic data is through a technique known as **data augmentation**. To take a concrete example from machine learning, consider the training of a neural network for the classification of images. Data augmentation implies that we create new images by, e.g., rotating, reflecting, cropping or translating existing images. This process is used to extend and diversify the training data, reducing over-fitting, which can occur when there is insufficient training data.

We provide a practical example of the use of Rule 6 and Rule 7 in the supplementary material (Appendix B in S1 File).

## Rule 8: Incorporate additional knowledge in your AI models

Machine-learning approaches are powerful since they can analyse vast quantities of data much more quickly than traditional methods without needing knowledge of the physical processes inherent within the data. Machine-learning approaches can also learn directly from data, meaning that we do not need to manually search for features or even understand what is expressed within the data. However, in many cases, incorporating any additional understanding of the data within machine-learning models can increase the robustness and trustworthiness of the model predictions and result in more stable predictions for a greater range of scenarios. This understanding can come from several main sources [29], including physical laws, symmetries, the rules of logic and knowledge graphs. For instance, if the data represent physical processes that are governed by particular equations, these equations can be included in the optimisation process performed during training [30]. If solutions are known to possess certain symmetries or to obey particular rules of logic, these properties can also be embedded in a neural network [31–34]. Including prior understanding in this way can also reduce the amount of data required to train machine-learning models [29].

For some approaches, such as the causal inference techniques outlined in Rule 1, incorporating your mechanistic understanding of the system into the model might very well form your starting point. For these methods, physical laws, symmetries and other knowledge thus enter from the very beginning. However, for these methods, any additional prior knowledge that you can use to improve any parameter inference tasks might come to bear and should be reconsidered after the initial development of your code.

## Rule 9: Aim for an explainable, interpretable and trustworthy AI model

There is an increasing desire to instil **explainability** and **interpretability** into AI models so that they can be shown to be fair, robust and trustworthy. For sectors such as energy, health, recruitment, and finance, it is crucial that the decisions or predictions of these models can stand up to scrutiny, as they may affect energy security, people’s medical treatments, job prospects and mortgage applications, for example. Governments and international organisations are publishing strategies and guidelines on how to achieve explainable, interpretable, trustworthy and fair AI algorithms [35–38].

For various machine-learning approaches, one strategy is to highlight which input features were most influential in a particular decision or prediction. Examples of methods that can do this include the **SHAP model**, which has been used to analyse the predictions made by machine-learning models for wildfires [39,40], and **saliency maps**, which have been used to analyse the classifications of images [41]. For neural networks in particular, one might wish to go further and attempt to understand the inner workings of the network by looking at the values or **activations** of its neurons. An example of such an approach is the beautiful activation atlas of Carter *et al*. [42], which attempts to visualise what images or inputs might cause a particular set of activations. Despite advancements in this area, there remains ambiguity as to what level of interpretability is possible and how to reconcile multiple interpretations of the same model suggested by different methods. Some are calling for broader approaches that could consider the societal factors that have influenced the model [43].

Achieving explainable and interpretable models can also contribute towards model reproducibility by diagnosing inconsistencies in model behaviour. Although highly desirable from both ethical and scientific points of view, reproducibility can be challenging for AI models due to a number of reasons, including variability arising from **random seeds**, the challenges of obtaining enough compute power to train someone else’s model, hardware-specific behaviour, and model sensitivity to noise. The first two issues could be alleviated by making models and their parameters and hyperparameters available (see Rule 5), although this solution does not guarantee bitwise identical outputs.

When undertaking AI-driven research, it is essential to actively consider these challenges associated with explainability, interpretability, and reproducibility to ensure scientific rigour and to foster a responsible research culture. Since our paper aims to provide concise rules for the application of AI, we have naturally had to set aside many specific nuances of AI ethics. We encourage the readers to explore this topic further (e.g., [44]).

## Rule 10: Keep your AIm in mind

Do not lose yourself in AI. As a scientist, it is important to maintain a balanced perspective: While AI can be incredibly powerful and offers many benefits, it is crucial not to become overly focused on optimising AI models at the expense of scientific rigour and understanding.

Whether you are developing AI methods to advance a scientific field or using them for specific tasks, it is easy to become absorbed in AI. For instance, in medical diagnosis, prioritising a model’s accuracy over interpretability can result in predictions that are difficult for clinicians to trust and act upon. Using interpretable models, even if they are slightly less accurate, can improve clinical decision-making and patient outcomes.

It is crucial to balance metric optimisation with scientific rigour, ensuring that the insights gained contribute meaningfully to broader understanding and real-world applications [45]. Additionally, the model’s generalisability beyond its training distribution must be carefully examined, as distributions often change over time, leading to mismatches between training and deployment. This is common for models trained on simulations but deployed on real data. Techniques such as **domain adaptation** and post-calibration related to transfer learning can help mitigate this issue.

Moreover, deriving statistical ( **aleatoric**) and systematic ( **epistemic**) uncertainties in the model predictions is critical and should be given high priority. Indeed, quantifying uncertainties is essential for making AI results scientifically interpretable. While a comprehensive discussion of model uncertainties and statistics is beyond the scope of this paper, we encourage readers to explore and address these topics when developing and deploying AI (see also [15,18]).

## Discussion

There is a growing body of literature on the impact of AI on scientific understanding and its pitfalls. While AI holds great promise for accelerating scientific research and discovery, it can often create an illusion of understanding that impedes true scientific progress [46,47]. We encourage readers to keep this in mind and strive for a mindful and explainable use of AI in science.

Moreover, the development of AI algorithms and changes in terminology are progressing quickly, leading to a pressure to stay up-to-date. When pursuing the cutting edge of AI applications in science, it is thus important to do so with a critical mindset regarding the robustness of methods and results (cf. Rules 9 and 10). We also note that the rapid pace of development in AI implies that specific AI packages or libraries might become outdated or fall out of fashion, including the examples listed in this paper.

For brevity, we have naturally also had to be selective about the presented concepts and examples. To address some of these topics, additional terms are listed in the glossary. While our ten rules for navigating AI do not cover every challenge you may encounter as a scientist working with AI, we hope they provide valuable guidance on your journey. By following these principles, you will be well-equipped to navigate the complexities ahead.

## Supporting information

S1 File Supplementary text and figures. Split into two appendices. Appendix A includes a glossary of key terms highlighted in bold throughout the paper. Appendix B covers a practical example of the use of Rules 6 and 7.
(PDF)
