# Supplementary material for: Ten simple rules for navigating AI in science
Source: PLoS Comput Biol. 2025 Jul 18;21(7):e1013259. doi: 10.1371/journal.pcbi.1013259 (PMC12273916; doi:10.1371/journal.pcbi.1013259)
Supplement: S1 File — Split into two appendices. Appendix A includes a glossary of key terms highlighted in bold throughout the paper. Appendix B covers a practical example of the use of Rules 6 and 7. (PDF) [file pcbi.1013259.s001.pdf]

A Glossary

Table A.1: The terms listed in this table are highlighted in bold in the corresponding sections.

**Term**	**Explanation**	**Associated rule**
**Accuracy**	A performance metric that measures the proportion of correct predictions made by the model. In binary classification tasks, this amounts to the fraction of correctly attributed labels.	Rule 1
**Activation**	For neural networks, activation is the output of a neuron, calculated from a weighted sum of the inputs to that neuron which is passed through an activation function. See Fig A.1.	Rule 9
**Activation function**	For neural networks, an activation function is applied to the weighted sum of the inputs to a neuron (or node) in order to evaluate that neuron's output or activation. It is often a nonlinear function. See Fig A.1.	Glossary
**Aleatoric vs. epistemic uncertainty**	We might distinguish between two different contributions to uncertainty. Aleatoric uncertainty is inherent to the precision of the experiment itself and thus results from the stochastic nature of the process in question. Meanwhile, our model might not fully capture the process, neglecting certain aspects, or measurements might not be accurate. Uncertainties that arise from such inadequacies are epistemic and could be addressed by improving the data collection or model.	Rule 10
**Approximate Bayesian Computation (ABC)**	ABC denotes a set algorithms used for Likelihood-free inference.	Fig 1
**Artificial Intelligence (AI)**	In this paper, we use the term AI in its most general sense. It thus covers different computational techniques, including machine learning and beyond. It has been defined to be "the science and engineering of making intelligent machines, especially intelligent computer programs" [1]. Generative AI, such as LLM, is a special type of deep learning, which is a subset of machine learning, and machine learning is, in turn, a subset of AI methods (see Fig 1.	Introduction
**Back-propagation**	For neural networks, it is an algorithm used in supervised learning tasks for efficient calculation of the gradient of the loss function with respect to the network's weights and biases [2].	Glossary
**Bias**	It is the difference (error) between the real-world values and the model-predicted values. A high bias means the model is not complex enough to encapsulate the data patterns (**underfitting**).	Rule 2

**Bias (parameters in neural networks)**	These parameters, just like weights, are updated at each **back-propagation** iteration in all hidden and output layers and help the model learn. See Fig A.1.	Rule 2
**Biased dataset**	A dataset is biased (unbalanced) when it systematically favours specific outcomes or groups.	Rule 2
**Bias-variance trade-off**	See **Bias** and **variance**. While having a low bias and low variance model is the objective, achieving both is often tricky. A simple model (e.g. linear regression) is often good at adapting to new data at the expense of failing to fit the training data exactly (high bias, low variance). On the other hand, more complex models (e.g. deep neural networks) may fit the training data better but do not generalise well to unseen data (low bias, high variance).	Rule 2
**Causal inference**	Causal inference attempts to establish whether changes in one variable lead to changes in other variables, i.e., it investigates causal relationships.	Rule 1
**Classification**	A supervised learning task where the output is categorical. This approach can be used to classify images into different categories.	Rule 1
**Clustering**	Approach to combine a set of unlabeled data points into meaningful subgroups (clusters) based solely on the structure contained in the data.	Rule 1
**Confounding factors**	In statistics, a confounder is a variable that might introduce spurious relationships by impacting both independent and dependent variables.	Rule 1
**Containerization**	Nowadays, machine-learning projects often build on existing packages (cf. Rule 4). As a result, you might wish to deploy your code in an isolated environment, a so-called container, with specific versions of packages and configurations.	Fig 2
**Data Augmentation**	A technique used to generate new samples based on existing data. In machine learning, data augmentation can be used to extend and diversify the training data set. For instance, when dealing with images, synthetic data can be created by reflecting, rotating, translating, cropping or adding noise to pre-existing images in the training dataset.	Rule 7
**Data cleaning**	Data cleaning is the process of identifying corrupt or inaccurate entries from a dataset. Once identified, the data points are modified or deleted.	Rule 1

**Data leakage**	In machine learning, data leakage describes processes where information is used to train the model that would not be available during prediction. In data privacy, data leakage refers to the unintentional exposure of data.	Glossary
**Deep Learning**	The field of **neural networks** and hence a subset of machine learning.	Introduction
**Dimensionality reduction**	Techniques that simplify high-dimensional data with a high number of features into a lower-dimensional representation with a smaller number of features. During this procedure, these methods seek to reduce the dimensionality without incurring a significant loss of important structures or patterns. The resulting lower-dimensional data can then be better visualised or combined with other unsupervised approaches, such as clustering.	Rule 1
**Domain adaptation**	Describes the improvement of model performance on a domain (target distribution) by using a model trained on a different domain, i.e., another distribution with the *same* feature space, for which more data is available.	Rule 10
**Explainability**	In machine learning, an explainable neural network is one whose output can be explained "in human terms" by knowledge of the input. For example, if a neural network model had declined an application for a mortgage, an explainable model would also be able to offer the reason for this (such as a low credit score or insufficient income). The term is sometimes used interchangeably with interpretability, although here, we differentiate between the two.	Rule 9
**FAIR**	Findable, Accessible, Interoperable, Reusable.	Rule 5
**Features**	Individual measurable properties or characteristics extracted from the data that are used by the algorithm to make predictions or decisions. For example, in an algorithm predicting crop yield, features could be temperature, soil type, fertiliser usage etc.	Glossary
**Foundation models**	Large-scale versatile machine-learning models trained on extensive and diverse data. They can be used for many tasks without retraining or with minimal and specific fine-tuning.	Rule 3
**Frozen weights**	Keeping certain weights of a model unchanged during the training process, which allows the model to retain previously learned information while adapting to new tasks. Particularly used in transfer learning.	Rule 3

**Generative AI**	Generative AI or generative algorithms are designed to capture the probability distribution underlying a particular training dataset. When inferring, the aim is to generate "new" examples that do not exist in the training dataset yet seem as if they belong to its distribution.	Introduction
**Hyper-parameters**	In machine learning, hyper-parameters denote aspects of a model which are set *a priori* and not learned, e.g. the architecture of a neural network. Note that it is still possible to explore these aspects by comparing models with different hyper-parameters. When training machine-learning models, you should track your hyperparameters. For this, we recommend Weights & Biases. In Bayesian hierarchical modelling, hyper-parameters are parameters related to the prior distribution.	Rule 6
**Interpretability**	In machine learning, interpretability is the ability to interpret the weights and features of a neural network. An interpretable neural network is more transparent to some degree, and questions can be addressed relating to how and why such a model behaves as it does. The term is sometimes used interchangeably with explainability, although here, we differentiate between the two.	Rule 9
**Labelled dataset**	A dataset in which each input example is paired with the correct output. The labels can be obtained, for example, by manual or automatic annotation of inputs by experts or by taking the parameters used to generate synthetic data.	Rule 1
**Large Language Models (LLMs)**	LLMs are AI algorithms for natural language processing (NLP) used for generating and understanding human language and are trained on large amounts of data.	Introduction
**Learning bias**	Inductive or learning bias is the set of assumptions a machine-learning algorithm makes to generalise from the training data to new data. These biases guide the model in making predictions, helping it generalise effectively, but they also limit the types of patterns the model can learn.	Rule 2
**Likelihood-free inference (LFI)**	For many complex real-world problems, it is not possible to state the likelihood in a closed form. The likelihood is intractable. LFI overcomes this issue by introducing a proxy for the likelihood based on the outcome of simulations.	Fig 1
**Library**	Compilation of existing code/functionality.	Rule 6
**Machine Learning**	A branch of AI in which predictive algorithms learn to generalise from training examples. Includes deep learning.	Introduction

**Machine Learning Operations (MLOps)**	Practices that automate and simplify machine-learning (ML) workflows and deployments.	Rule 5
**Model zoos**	A collection of pre-trained AI models available for download and use, enabling direct application or fine-tuning for transfer learning. Examples include the BioImage Model Zoo for microscopy images [3], the Ecoviz Model zoo for ecology tasks, the Koopman Model zoo for Koopman operators, or Kipoi [4] for genomics.	Rule 3
**Modular functions**	In coding, functions are portions of code which take inputs, apply operations and return outputs. Modular functions are those which are written to be flexible and so reusable.	Rule 4
**Neural Network**	Inspired by neuroscience, neural networks are models used for **deep learning**, which process data for various tasks through layers of units or neurons. Neurons are interconnected through weights that can be learned using optimisation techniques. (See Fig A.1.)	Rule 2
**Overfitting**	Overfitting occurs when a model is too specific to the data it has been trained on and does not generalise well to new data. This results in high **variance**, with high performance on training data but poor performance on test data.	Glossary
**Parameters**	A parameter is a numerical value that characterises a system or model. In the context of machine learning, parameters include the weights and biases in a neural network or the coefficients in a regression model, all of which are learned from data during training. See Fig A.1.	Rule 6
**Random forest (RF)**	Supervised machine-learning algorithm drawing on multiple decision trees, combining their predictions.	Rule 6
**Random seed**	A number used to initialise algorithms for generating random numbers. Computer algorithms that generate random number sequences are not truly random. Rather the sequences follow from the initial conditions. By fixing a random seed, the same random sequence can be recovered when rerunning the code.	Rule 9
**Regression**	A **supervised learning** task where the output is continuous.	Rule 1
**Reinforcement Learning**	Reinforcement Learning is a method in which an autonomous agent learns how to perform a task by trial-and-error using feedback from its actions. It is one of three main paradigms of machine learning, alongside **supervised** and **unsupervised** learning.	Introduction

**Saliency Maps**	For a machine-learning classification task, a saliency map is an image that highlights which regions influence how the network classifies the image. The map is found by calculating the sensitivity of the class scores to each pixel using backpropagation.	Rule 9
**Sequential Monte Carlo (particle filters)**	A technique that approximates probability distributions over time using weighted samples or particles. It is well suited for dynamic systems with sequential state evolution. The algorithm continuously updates particles with new data and resamples them based on their likelihood of representing the correct state.	Rule 2
**SHAP model**	**SH**apley **A**dditive ex**P**lanations is an approach based on game theory which can be used to explain the output of any machine-learning model by calculating how much each input contributes to the model's output.	Rule 9
**Simulation-based inference**	Set of statistical techniques where simulations help estimate parameters or make predictions. A model generates synthetic data under different parameter configurations, and these simulated outcomes can be compared to real data.	Fig 1
**Supervised Learning**	Algorithm trained to map labelled inputs to the corresponding outputs by using features from the data. After training, the algorithm can make predictions on new, unseen data based on the mapping it has learned. Supervised learning is commonly used in regression and classification tasks.	Rule 1
**Support Vector Machines (SVM)**	Supervised classification algorithm that aims to create a decision boundary that separates classes by maximising the distance between the decision boundary and near points in each class.	Rule 6
**Synthetic data**	Data generated artificially - such as by sampling from distributions or created through simulations.	Rule 6
**Testing**	Evaluating a model's performance using data which was not used during training (termed the test dataset).	Rule 6
**Training**	In machine learning, the term training denotes the use a dataset (termed the training dataset) to fit a model's parameters.	Rule 6
**Transfer learning**	Machine-learning technique aiming to repurpose a model trained for one task into one trained for a related task. This can be done directly or after some fine-tuning, decreasing the amount of data and computational power required for the original task.	Rule 3

**Underfitting**	Underfitting occurs when a model does not represent the data it has been trained on well and does not give accurate results. This results in high **bias**, with poor training and test data performance. For instance, depending on the considered regime, a second-order polynomial will not be able to capture data created using a sinusoidal function very well.	Glossary
**Unsupervised Learning**	Algorithm trained on data without labels. The goal of unsupervised learning is to identify patterns, structures, relationships or optimal clustering directly from the data.	Rule 1
**Variance**	An error introduced by the model's sensitivity to small fluctuations in the training data. It represents how much predictions would change if the model were trained on a different dataset. A model with high variance is too specific to the training data, including noise or irrelevant details (**overfitting**).	Rule 2
**Weights**	Values in a model that determine the importance of each feature in predicting the target output. The model adjusts these weights during training to decrease the error between its predictions and the target output. See Fig A.1.	Rule 3

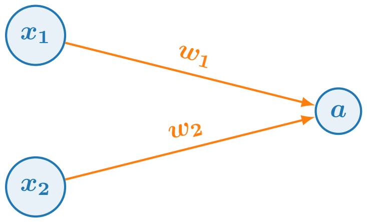

$$a = \sigma\left(\sum_i w_i x_i + b\right)$$

Figure A.1: Two (input) neurons with values x_1 and x_2 connected to a third neuron with a value or activation of a. The weights are given by w_1 and w_2, and the bias is b. The activation function is represented by σ.

References

[1] John McCarthy. What is AI? https://www-formal.stanford.edu/jmc/whatisai.pdf. Accessed: 09-09-2024.

[2] Yann LeCun, Yoshua Bengio, and Geoffrey Hinton. Deep learning. *Nature*, 521(7553):436–444, 2015.

[3] Wei Ouyang, Fynn Beuttenmueller, Estibaliz Gómez-de Mariscal, Constantin Pape, Tom Burke, Carlos Garcia-López-de Haro, Craig Russell, Lucía Moya-Sans, Cristina de-la Torre-Gutiérrez, Deborah Schmidt, Dominik Kutra, Maksim Novikov, Martin Weigert, Uwe Schmidt, Peter Bankhead, Guillaume Jacquemet, Daniel Sage, Ricardo Henriques, Arrate Muñoz-Barrutia, Emma Lundberg, Florian Jug, and Anna Kreshuk. Bioimage model zoo: A community-driven resource for accessible deep learning in bioimage analysis. *bioRxiv*, 2022.

[4] Ž. Avsec, R. Kreuzhuber, J. Israeli, N. Xu, J. Cheng, A. Shrikumar, A. Banerjee, D. S. Kim, T. Beier, L. Urban, A. Kundaje, O. Stegle, and J. Gagneur. The kipoi repository accelerates community exchange and reuse of predictive models for genomics. *Nature Biotechnology*, 37:592–600, 2019.

B Worked example

Imagine being given a dataset with ten features from 100 samples, 70 of which come from group A and 30 from group B — this is our training set. Your goal is to use this training set to build a model that can assign or classify new samples into groups A/B as accurately as possible. For this purpose, you withhold some data for which the correct classification is known for final tests — this is your test data. Only if you can convince yourself that your model reliably classifies seen and unseen examples can you trust your model to be put into use and actually attribute labels to data. Notably, you want to withhold the test data until your final evaluation. You should thus not reuse the test data for model selection, as this would lead to overfitting. To prevent this, it is common practice to split the training set further into a training and a validation set. The validation set is then used for intermediate testing during the model development process.

Following Rule 6 and 7, you should

1. Establish a baseline, which tells you how well you can perform by ignoring the features and simply guessing the class. For equally sized groups (known as balanced classes), this will be $100/n$, where n is your number of classes. For unbalanced classes, you should use the percentage of data from the most common class (this is known as a majority class classifier). In our case, any model with performance $< 70\%$ accuracy should be ignored.

2. Try a non-deep learning approach: regression for predicting continuous variables or a random forest classifier for discrete classes. Let's say the latter model achieves 80% accuracy on our test set; this is above our baseline and may already be sufficient for our needs.

3. If not, start training and testing small, feed-forward neural networks and build complexity from there.

Generate Synthetic Data

```
import numpy as np
def generate_synthetic_data(n_samples_per_class, n_features, mean_per_class):
"""
Generates synthetic data by drawing from normal distributions.
Arguments:
n_samples_per_class: a list with the number of samples per class.
n_features: an integer with the number of features to generate.
mean_per_class: a list with the mean value per class.
The more similar these are, the harder the problem will be.
Returns:
data: a samples x features np array.
class_labels: a np vector with an integer label per class.
"""
assert len(n_samples_per_class) == len(mean_per_class), "Each class requires a mean value"
data, class_labels = [], []
for class_label, n_samples in enumerate(n_samples_per_class):
    data.append(np.random.normal(loc=mean_per_class[class_label],
    scale=1.0,
    size=(n_samples, n_features)))
    class_labels.append(np.repeat(class_label, n_samples))
return np.concatenate(data), np.concatenate(class_labels)
```

To ensure that these models are working, we recommend that you apply the model to synthetic data. For

example, one could generate n samples per class by drawing from a normal distribution with a different mean per class. In Python, we could achieve this with a simple function, as shown in the code snippet above.

If your code fails to perform well given synthetic data like these, with reasonably different mean values per class, it is unlikely to solve a real-world problem in which the data will be noisier. Try checking and changing your code until it can solve simple synthetic problems like these.

The advantage of starting with synthetic data is that it allows you to adjust the complexity of your tests while knowing the underlying ground truth (cf. Rule 7). Moreover, for many systems, sophisticated simulations effectively capture the corresponding real-world phenomena and can generate synthetic data that closely mimic actual data — in such cases, simulations may even serve as your training data, as your aim may be to emulate the output of the simulations to speed up computations. Meanwhile, for many other systems, it may be impractical to approximate the generative process behind the data, or such models may not be available. As stated above, even in those cases, you might still learn valuable lessons and spot bugs based on synthetic data from toy models.
